# Supplementary material for: Efficient and continuous microwave photoconversion in hybrid cavity-semiconductor nanowire double quantum dot diodes
Source: Nat Commun. 2021 Aug 26;12:5130. doi: 10.1038/s41467-021-25446-1 (PMC8390526; doi:10.1038/s41467-021-25446-1)
Supplement: Supplementary file 1 — Supplementary Information [file 41467_2021_25446_MOESM1_ESM.pdf]

Supplementary Information - Efficient and Continuous Microwave Photoconversion in Hybrid Cavity-Semiconductor Nanowire Double Quantum Dot Diodes

W. Khan,[1] P. P. Potts,[2] S. Lehmann,[1] C. Thelander,[1] K. A. Dick,[1,3] P. Samuelsson,[2] and V. F. Maisi[1,*]

[1]*NanoLund and Solid State Physics, Lund University, Box 118, 22100 Lund, Sweden*
[2]*NanoLund and mathematical Physics, Lund University, Box 118, 22100 Lund, Sweden*
[3]*Center for Analysis and Synthesis, Lund University, S-221 00 Lund, Sweden*

(Dated: August 10, 2021)

* ville.maisi@ftf.lth.se

SUPPLEMENTARY METHODS

Determination of the directivity term and DQD energies

The directivity of the DQD photodetector is given by the term $p_f = \cos(\theta) = \delta_r/hf_r = \sqrt{1-(2t/hf_r)^2}$, where θ is the so-called mixing angle, δ_r the detuning at resonance, t the interdot tunnel coupling and f_r the resonance frequency of the resonator [1, 2]. To determine the directivity, we use the value $f_r = 6.436\,\mathrm{GHz}$ obtained from the resonator response. To obtain the other parameter value, the detuning δ_r in resonance, we first determine how the left gate electrode changes the detuning δ based on finite bias transport measurements. Then based on identifying the detuning when the DQD is in resonance with resonator yields us both the detuning and the interdot coupling t in the photodetector operation point. We present below the measurements and the procedure of these experiments in detail.

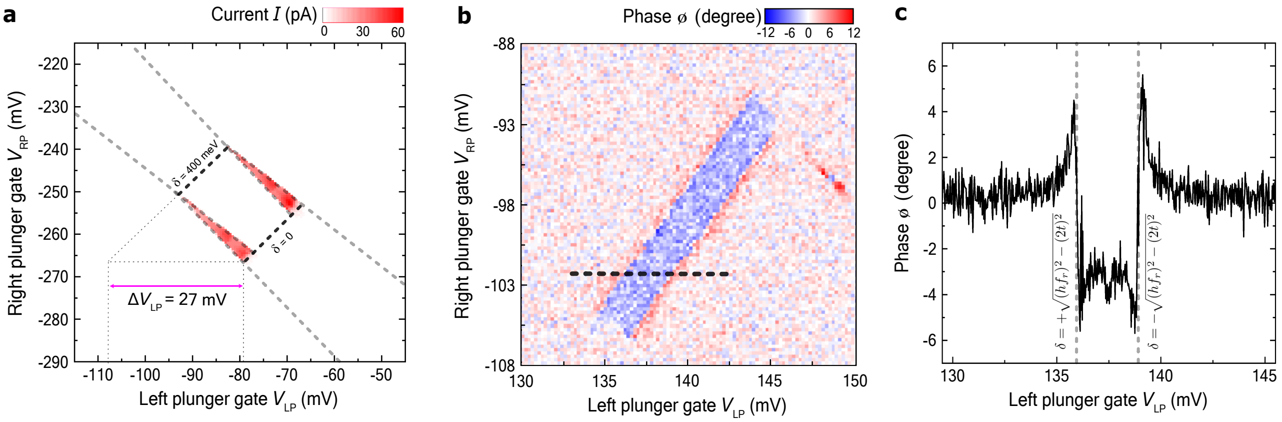

SUPPLEMENTARY FIGURE 1. **a**, A pair of finite bias triangles from the charge stability diagram of DQD at $V_B = 400\,\mu\mathrm{V}$. **b**, Phase response at interdot charge transition regime as a function of plunger gate voltages V_{LP} and V_{RP}. **c**, Phase response cut through the interdot charge transition (dashed line in panel b). The DQD had a few charge reconfigurations between the measurements (sudden jumps in the gate voltages). By measuring the finite bias triangles (fingerprint from excited states and the electrical current I profile), we assured that we were investigating the same transition at all times.

In Supplementary Fig. 1a, we present a finite bias measurement of the DQD. The bias voltage is set to $V_B = 400\,\mu\mathrm{V}$ via the resonator middle contact. From the finite bias triangles, we identify two detuning conditions [3]: at the base of the triangles the detuning vanishes, that is $\delta = 0$ and the peaks of the two neighboring triangles define a detuning line corresponding to bias voltage, i.e. $\delta = eV_B = 400\,\mu\mathrm{eV}$. These values are the minimum and maximum detuning values such that the energy levels are within the transport window and dc current flows. Since the gate voltages move the energy levels of the quantum dots up and down with a linear relation, measuring the distance in the left gate voltage direction denoted as ΔV_{LP} in the figure, yields us the lever arm $\alpha_{LP,\delta} = eV_B/\Delta V_{LP} = 400\,\mu\mathrm{eV}/27\,\mathrm{mV} = 14.8\,\mu\mathrm{eV/mV}$. This lever arm characterizes how much the left gate voltage shifts the detuning δ of the DQD. Here we have neglected the interdot tunnel coupling t contribution to the total energy $E(\delta) = \sqrt{\delta^2 + (2t)^2} \approx |\delta|$. This approximation is well justified since $eV_B \gg t$ as we will see next.

Now we determine the δ_r and the interdot tunnel coupling based on the resonance condition $E(\delta_r) = hf_r$. Here we use the full expression $E(\delta) = \sqrt{\delta^2 + (2t)^2}$ without the above approximation since with the resonator phase response to the DQD we are able to measure the small deviance that t causes to the approximate relation used elsewhere in our data analysis. Supplementary Figures 1b and c, present the phase response of the resonator as a function of the DQD gates. We observe a response along the direction where the detuning changes while the response stays constant along the perpendicular direction similarly as in previous experiments [4–6]. We observe the resonance of the DQD with the microwave resonator as a sharp transition in the phase response. We have one resonance for positive detuning and one for negative detuning. Panel c shows a cut of the response with the left gate voltage V_{LP}. With that, we measure the distance of the positive and negative resonance condition $\Delta V_{LP} = 2.84\,\mathrm{mV}$. The resonance conditions $E(\pm\delta_r) = hf_r$ result in a sharp transitions from a positive phase to a negative in the phase response. With the above lever arm, we

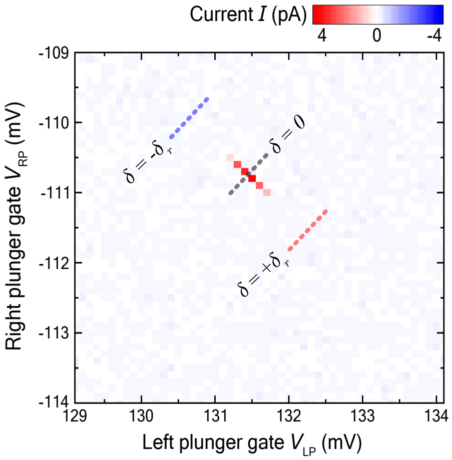

SUPPLEMENTARY FIGURE 2. Photodetector response without applied microwave signal. No measurable signal is observed at the photodetector points. Electrical current flows only at a minuscule area around the triple point with $\delta = 0$.

convert this to the detuning at the resonance $\delta_r = \alpha_{LP,\delta}\, \Delta V_{LP}/2 = 14.8\,\mu\text{eV/mV} \cdot 2.84\,\text{mV}/2 = 21.0\,\mu\text{eV}$ yielding us the directivity $p_f = 0.79$. With the total energy difference of the DQD in the resonance, $E(\delta_r) = \sqrt{\delta_r^2 + (2t)^2} = hf_r$, we also obtain the tunnel coupling as $t = \sqrt{(hf_r)^2 - \delta_r^2}/2 = 8.1\,\mu\text{eV}$.

In Supplementary Fig. 1a, we see the first excited state with pronounced current in the middle of the triangles. The bias voltage opens up an energy window of 400 µeV. As the excited state is in the middle of it, we estimate the higher excited states of the quantum dots to be approximately 200 µeV above the states considered for the photodetector. Since this energy is an order of magnitude larger than the photon energy, and other energies in the system such as the thermal energy $k_B T$, the excited states of the quantum dots do not influence the photodetector operation.

Similar to the above determination of the detuning energies, we also determine the energy shifts along the detuning axis. This direction is identified by the base of the finite bias triangle where the energy difference E of the ground and excited state is constant and the states are move up or down in energy in concert. The length of the triangle base in Supplementary Fig. 1a at $\delta = 0$ corresponds to an energy shift of 400 µeV of the DQD energy levels. This allows us to determine the source-drain energy window of $E = hf_r$ presented in Fig. 3a of the main manuscript.

Dark current

Supplementary Figure 2 presents the photodetector response with no applied microwave signal. We observe a miniscule signal at the charge triple point at $\delta = 0$. At the photodetector points $\delta = \pm\delta_r$, we see no measurable current. Thus, the dark current of the photodetector is below the 0.2 pA noise level of the measurement.

Charge configuration

All the measurements presented in the article and in the Supplemental Material have been performed at the same charge configuration of the DQD. The plunger gate voltages vary from measurement to measurement due to slow drift of the offset charges (in a timescale of a week) that we followed during the course of the measurement. Supplementary Figure 3 summarises the measurements that we used for assuring to stay at the same operation point.

Supplementary Figure 3a presents an overview of the different charge numbers. Around each of the configurations, we observe a finite bias triangle pair with unique fingerprint. The magnitude of electrical current varies between each triangle with some having higher current at the base and other at the tip and some on the adjacent sides. Also, each one of them has a characteristic excited state spectrum forming lines along the triangles. The zoom-in of panel b presents the structure of the charge configuration we used throughout the manuscript at around $V_{\text{RP}} = -200\,\text{mV}$ and $V_{\text{LP}} = -10\,\text{mV}$. Over the course of the measurements, we followed this charge transition point and made control measurement to check that we have the same charge transition whenever the configuration changed considerably. Supplementary Figures 3c and 3e show examples of these together with the repetition of the photodetection measurement of Fig. 3a

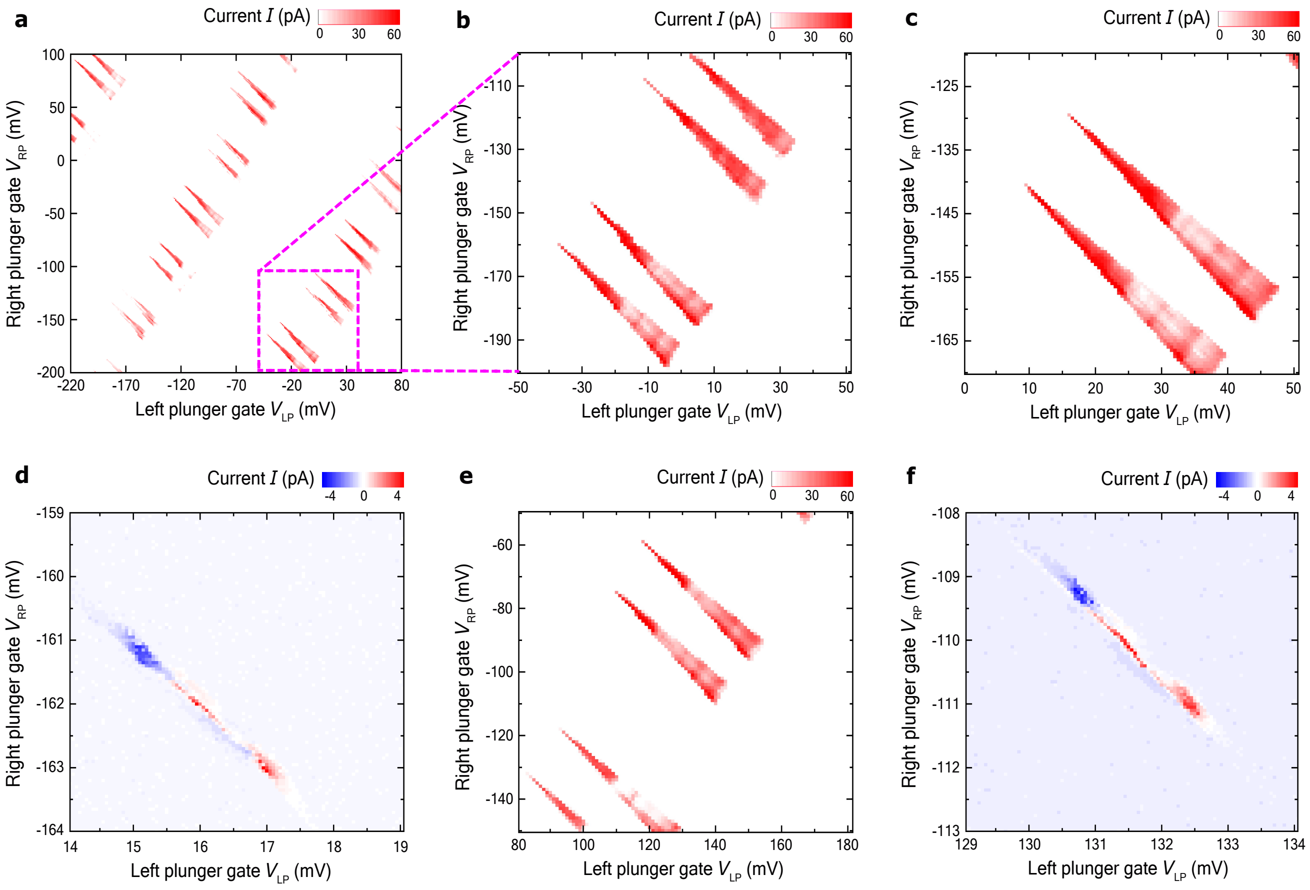

SUPPLEMENTARY FIGURE 3. **a**, Charge stability diagram of the DQD over larger gate voltage range with bias voltage $V_B = 1\,\mathrm{mV}$. **b**, Zoom-in of the diagram containing the selected charge configuration at the bottom-most triangle measured at day 35. The corresponding photodetection measurement is presented in Fig. 3a of the main article. **c**, The same measurement as in b but at day 14 when the configuration was at $V_{\mathrm{RP}} = -170\,\mathrm{mV}$ and $V_{\mathrm{LP}} = 35\,\mathrm{mV}$. **d**, Photo detection measurement similar to Fig. 3a of the main article done after the measurement in panel c. Panels **e** and **f** present a similar set of measurements carried out at day 1 which is the same day when the phase response measurement of Fig. 3b of the main manuscript was done. These three plots form a set of data where the charge stability diagram (panel **e** here), photocurrent (panel **f** here) and phase plot (panel 3b of the main article) are measured with the same gate voltages within a few mV drifts at most between the measurements.

of the main article in panels d and f correspondingly. We see the same features in the finite bias triangles as well as the same photoresponse at $\delta = \pm\delta_r$. Panels d and f have a stronger response to the positive current polarity only around $\delta = 0$ as compared to the data in Fig. 3a of the main article. This arises as in these measurements the offset voltage of the current pre-amplifier was not tuned as well as for the data in the main article. It's worth to note that the magnitude of the photoresponse at $\delta = \pm\delta_r$ is unaltered as it is not sensitive to small bias voltages, i.e. if the chemical potential of either source or drain shift a little in the schematic diagram of Fig. 1a in the main article. We cannot for sure identify the origin of the current reversing response at $\delta = 0$ of Fig. 3a in the main article. However, we speculate that it arises from a similar effect as reported in Ref. 7.

Determination of the microwave powers at the detector

The microwave signals were sent in and the outcoming signals measured with a standard heterodyne detection scheme [8]. The cabling in the cryostat is outlined in Supplementary Fig 4. Outside of the cryostat, the input signal from a RF generator was splitted into two parts. One of the signals was used as a phase reference mixed directly with a local oscillator (LO) and the second one was sent into the device via the cryostat and then mixed with the LO, digitized and then the amplitude and phase response was determined with digital signal processing by determining

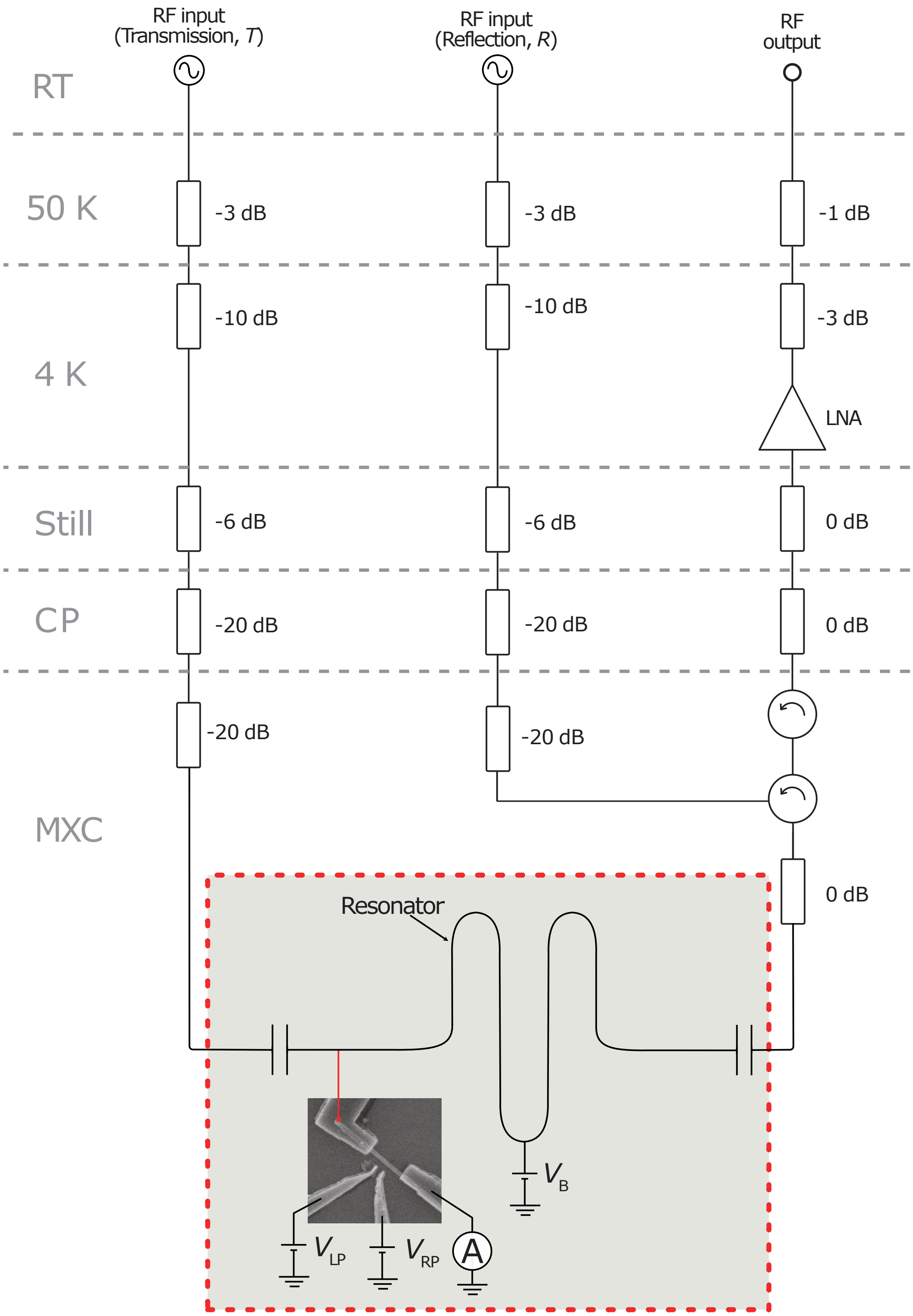

SUPPLEMENTARY FIGURE 4. Schematics of RF cables used for sending in microwave photons and measuring the microwave response.

the amplitudes and phases at the IF frequency.

To determine the in and out going microwave powers at the device, we need to know the microwave attenuation in

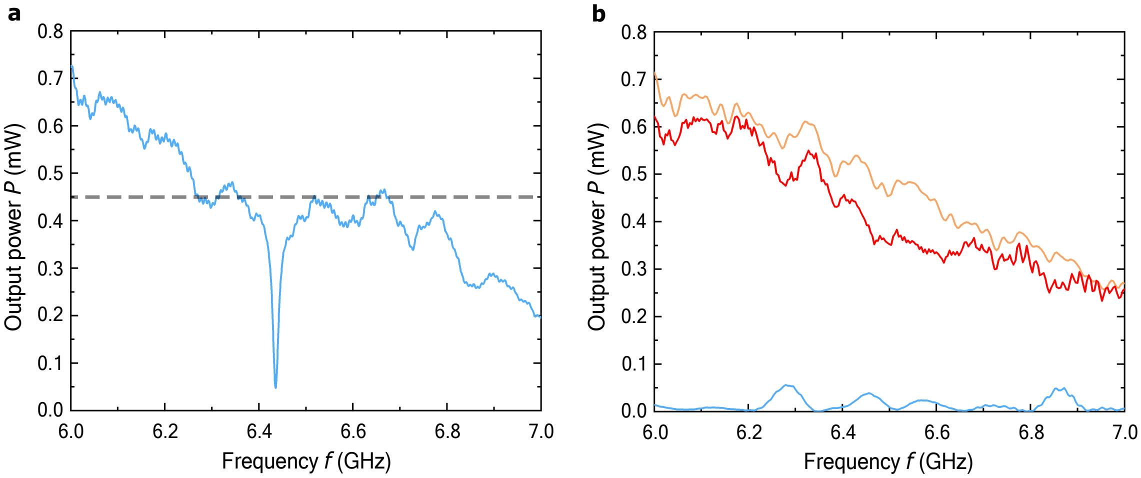

SUPPLEMENTARY FIGURE 5. **a**, Reflected output power P at the output signal digitizer with an input power of 10 mW at the signal generator. In the middle of the figure we see the resonator response and the dashed black line shows the calibration value of -3.5 dBm determined with the background level at $f = 6.3 - 6.6$ GHz. **b**, Output power P for the reflected (blue) and transmitted signal (red) for a direct transmission line mounted instead of the photodetector. The coral signal shows the reflected power with the sample holding probe completely removed. In that case we obtain somewhat higher signal than the red transmission data as two 30 cm long copper lines and the circuit board holding the device are not part of the signal chain as they are removed with the sample holder.

the signal lines. Supplementary Figure 4 outlines the microwave lines, their attenuators, amplifiers and circulators at each temperature stage. The connecting lines between different temperature stages are nominally identical in all the three lines. In addition to the identical connecting lines, we have the components listed in Supplementary Table I. The input lines contain an extra cable, power splitter and attenuators in the cryostat. The output line, on the other hand, has a dual-circulator, cryogenic amplifier, attenuators but with lower losses, two room temperature (RT) amplifiers, mixer, intermediate frequency (IF) attenuation, amplifier and a low pass filter. We list the attenuation/gain for the components in the supplementary table, based on calibration datasheet (cryogenic amplifier) and the datasheet specifications (rest of the components). This leaves the attenuation of the cryostat cables x as the only unknown that is out of construction the same for all lines.

We determine x close to the measurement frequency by measuring the reflection coefficient well off from the resonance frequency f_r as presented in Supplementary Fig. 5a. The reflection coefficient off from the resonance is unity, i.e. all power is reflected back. By sending in a $P = 10$ dBm microwave signal and measuring a $P = -3.5$ dBm signal at the output yields us a total cable attenuation of 23.9 dB when accounting for the components of the Supplementary Table I. Since we have identical lines, half of these losses are at the input and half at the output and hence $x = -11.9$ dB. The total attenuation from signal generator to the device is hence 80 dB.

In addition, to the components listed above, spurious losses could potentially alter the energy balance. The most relevant spurious losses are:

- Extra connectors / interconnects in the output line for the circulator and amplifier: We use two Minicircuits 086-4SM+ cables for interconnects, one for the cryogenic amplifier and one for the circulator in the output port. These cables have an insertion loss of 0.2 dB at $f = 7$ GHz at room temperature. We anticipate that the losses at the operation temperature $T < 3$ K is lower than this as the metals get more conducting towards lower temperatures. Hence these have in total less than 0.4 dB (i.e. less than 10 % to the power balance) influence on the power balance calculation above. The extra SMA connectors are specified to have return loss that is better than 30 dB. Hence the mismatch arising from the extra connectors is negligible (less than 0.1 % to the power balance).

- Bonding wires: Bonding wires from the input and output ports to the printed circuit board connect the photodetector to the measurement setup. The bond wires can potentially cause attenuation and impedance mismatch to the circuit. To assess this, we measured a direct transmission line piece bonded instead of the photodetector to the measurement setup. The data is presented in Supplementary Fig. 5b. In this case we anticipate ideally full transmission and no reflected signal. We see that indeed, the transmitted signal in red follows the same background level as the fully reflected background signal in Supplementary Fig. 5a within 20 % or better. Also,

SUPPLEMENTARY TABLE I. Components and their attenuation or gain in the microwave lines.

Component	Attenuation/Gain at $f = 7$ GHz (dB)
**Input lines:**	
Output inter-connect cable, Minicircuits 086-36SM+	-1.84
Power splitter, Minicircuits ZX10R-14-S+	-7.25
Attenuators in the cryostat (Fig. 4)	-59.0
Cable losses	x
**Output line:**	
Cryogenic circulator, LNF-CIISC4_8A, Insertion loss	-0.2
Cryogenic amplifier, LNF-LNC4_8C	$+39.0$
Cable losses	x
Attenuators in the cryostat (Fig. 4)	-4.0
2 x RT amplifier, Minicircuits ZX60-83LN12+	40.4
Attenuators at RT	-6.0
Mixer, Minicircuits ZMX-10G+, Conversion loss	-4.76
IF attenuator	-3.0
IF amplifier, Femto DLPVA	$+20.0$
LP filter attenuation	-3.0

the reflected signal is correspondingly suppressed to 20 % or below. Also the reflected signal without the probe (full reflection expected for disconnected probe) presents the same background level with slightly increased amplitude due to two 30 cm cables removed from the signal chain leading to lower overall losses. With these findings, we conclude that bonding wires influence the gain calculation by less than 20 %. It is also worth noting that the two ports are symmetric - also in terms of bonding - and hence the symmetric part of the possible attenuation from the bond wires is accounted for with the above attenuation determination.

- Impedance mismatches: The input of the cryogenic amplifier has typically some amount of impedance mismatch that gives rise to part of the signal reflected back. Similarly, other components of the setup may cause reflections that give rise to standing waves. The setup is, however, build such that these standing waves are damped. For example for the input of the cryogenic amplifier, the reflected power goes to the circulators. They damp the reflected signal by 20 dB each making the reflected signals negligible. Similarly, the signal reflected from the input to the photodetector is damped by the attenuators of the input line. In the background signals presented in Supplementary Fig. 5 we observe that the oscillations arising from standing waves are below 20 % of the signal power. Similarly, the spuriously reflected signal of Supplementary Fig. 5b is below 20 % of the input signal. Note that here we have a frequency dependent attenuation that decreases the power towards higher frequencies, which is typical for microwave components. With these arguments, we estimate the total amount of the reflected signals in the measurement chain to be less than 20 %.

With the above analysis, the largest uncertainty in the power calibration arises from the 20 % variations of the background signal level. Therefore we estimate the power calibration to be correct within the 20 % accuracy, i.e. to about 1 dB. This uncertainty of the input power P appears directly as a uncertainty of the quantum efficiency η via Eq. (1) of the main article. With this, we obtain the error estimate $\eta = 6\ \% \pm 1\ \%$.

In the main article, we have considered the quantum efficiency η, i.e. the number of electrons passing the device per in-going photons to the input port. Alternatively, it is possible to consider the conversion factor between the measured current I and photon number in the cavity as those have a linear correspondence in the photodetector. Based on the theoretical model, at $P = 1$ fW, we have approximately 15 photons in the cavity with an electrical current of $I = 2.4$ pA. Hence each photon in the cavity contributes to an electrical current of 0.16 pA in the linear response regime.

[1] C. H. Wong and M. G. Vavilov, Phys. Rev. A **95**, 012325 (2017).
[2] L. Childress, A. S. Sørensen, and M. D. Lukin, Phys. Rev. A **69**, 042302 (2004).
[3] W. G. Van der Wiel, S. De Franceschi, J. M. Elzerman, T. Fujisawa, S. Tarucha, and L. P. Kouwenhoven, Rev. Mod. Phys. **75**, 1 (2002).
[4] T. Frey, P. Leek, M. Beck, A. Blais, T. Ihn, K. Ensslin, and A. Wallraff, Phys. Rev. Lett. **108**, 046807 (2012).
[5] H. Toida, T. Nakajima, and S. Komiyama, Phys. Rev. Lett. **110**, 066802 (2013).
[6] X. Mi, J. Cady, D. Zajac, P. Deelman, and J. R. Petta, Science **355**, 156 (2017).

[7] S. Cornia, F. Rossella, V. Demontis, V. Zannier, F. Beltram, L. Sorba, M. Affronte, and A. Ghirri, Sci. Rep. **9**, 19523 (2019).
[8] C. Lang, "Quantum microwave radiation and its interference characterized by correlation function measurements in circuit quantum electrodynamics," (PhD thesis, ETH Zurich, 2014).
